# Measurement for Change: From Idea to Approach

**DOI:** 10.3389/fpubh.2020.581756

**Published:** 2020-11-26

**Authors:** Lotte van der Haar, Penny A. Holding, Joachim Krapels, Joost de Laat, Wiedaad Slemming

**Affiliations:** ^1^Utrecht Centre for Global Challenges, Utrecht School of Economics, Utrecht University, Utrecht, Netherlands; ^2^Saving Brains Collaborative Learning Team, Bromyard, United Kingdom; ^3^Porticus Global, Amsterdam, Netherlands; ^4^Department of Pediatrics and Child Health, School of Clinical Medicine, University of the Witwatersrand, Johannesburg, South Africa

**Keywords:** implementation science, measurement for change, collaborative research, ECD, monitoring, evaluation and learning (MEL), intervention

## Abstract

Measurement for Change proposes an integration of monitoring, evaluation, and learning into decision-making systems that support sustainable transition of interventions to scale. It was developed using a cyclical, interactive 1-year dialogue between early childhood development (ECD) practitioners and academics from across the globe. Details are presented in Krapels et al. (1) as part of this special issue in Frontiers. In this paper, we trace the developments that inspired Measurement for Change and the novel ways in which the approach and the special issue was developed. The experience, and the reflections on this experience, are intended to inform those implementing initiatives that similarly seek to integrate practitioner- and academic experiences in support of sustainable transitions of interventions to scale.

Measurement for Change, in common with other current approaches to implementation science (2) recognizes the need for a more holistic, process based, adaptive, and learnings informed approach to implementation (1). This recognition is also central to three recent series in early childhood development (ECD), published in the Lancet (2016), the Annals of the New York Academy of Sciences (2018), and the Archives of Disease in Childhood (2019). These series highlight that strengthening monitoring, evaluation, and learning (MEL) systems are key in generating knowledge for effective intervention delivery and scale-up of these interventions with consistent effectiveness (3, 4).

The Measurement for Change approach hopes to contribute to the development of effective MEL systems for implementation and transitioning to scale. At the core of the Measurement for Change approach are five aspirations that can guide practitioners and researchers designing and using MEL systems in ECD programs, namely MEL systems that are (1) dynamic, (2) inclusive, (3) informative, (4) interactive, and (5) people-centered. Cross-cutting these five aspirations is a focus on human dignity and a human-rights centered approach. For details on the approach, see Krapels et al. (1) in this special issue. These aspirations serve to expand our thinking on why and how measurement, in its various ways and forms, can be utilized to create effective programs serving families and children.

These aspirations were arrived at through a structured dialogue over the course of a year between and among a group of academics and ECD practitioners representing 21 organizations working with families and young children in low resource settings across the globe. Most of the participants in this dialogue submitted individual contributions to this special issue, reflecting on MEL experiences in the context of their respective ECD initiatives. In this paper, we trace the developments that inspired this Measurement for Change approach, how the structured dialogue was organized, and reflect on this experience.

The progression from idea to approach is illustrated in Figure 1, which has as its center the four stages or components that guide our narrative. This model draws on the principles of a number of implementation frameworks. While each component informs the activities and reflections of the next, the process is not linear; the four components overlapped. When experiences suggested a need to revisit earlier components, adjustments were made to refine and improve the process.

**Figure 1:**
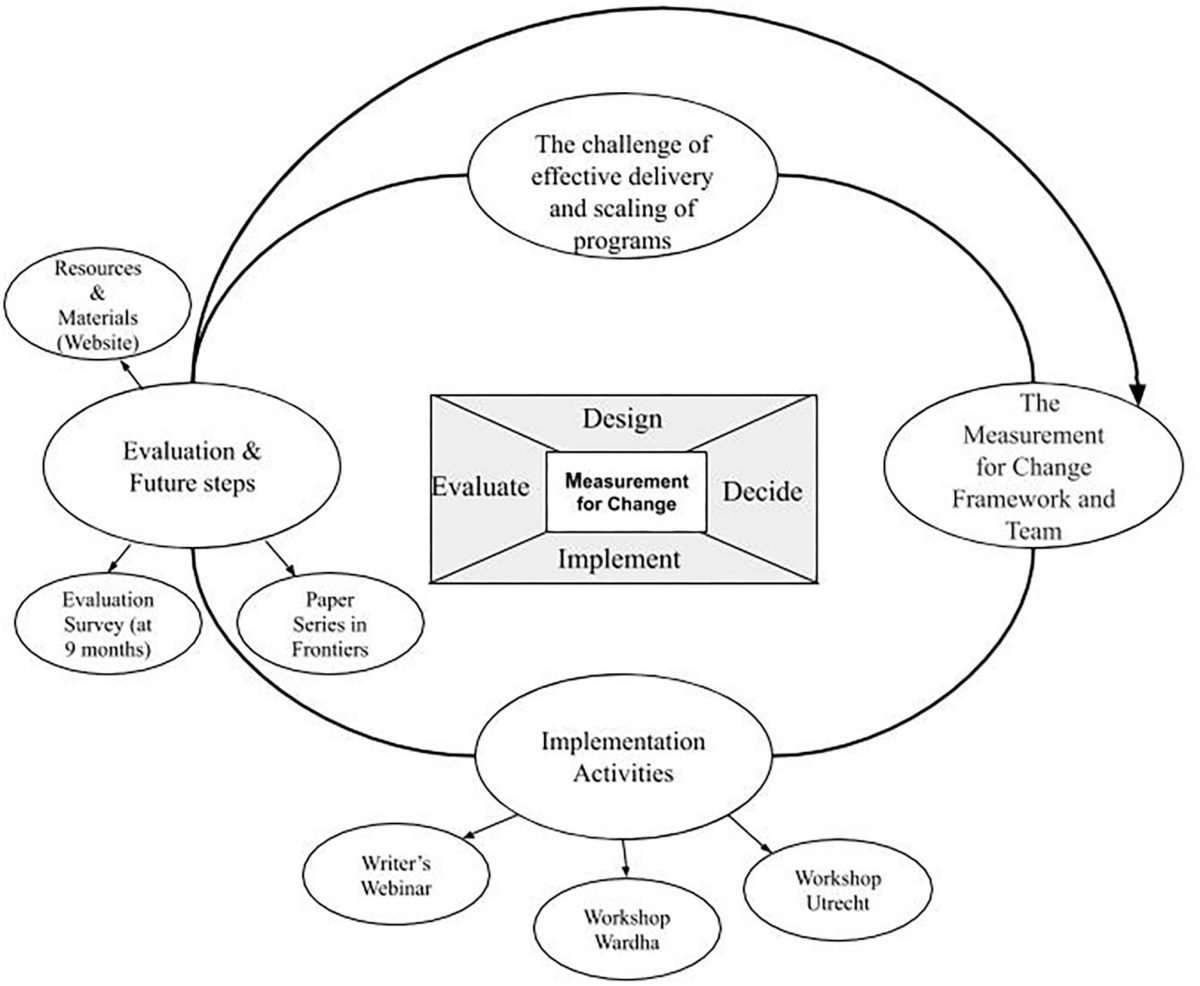
Development of Measurement for Change Approach.

## Design

Here, we outline the influences that culminated in the decision to put together this special issue.

### Evolution in the Measurement of Complex Systems

**Figure 2:**
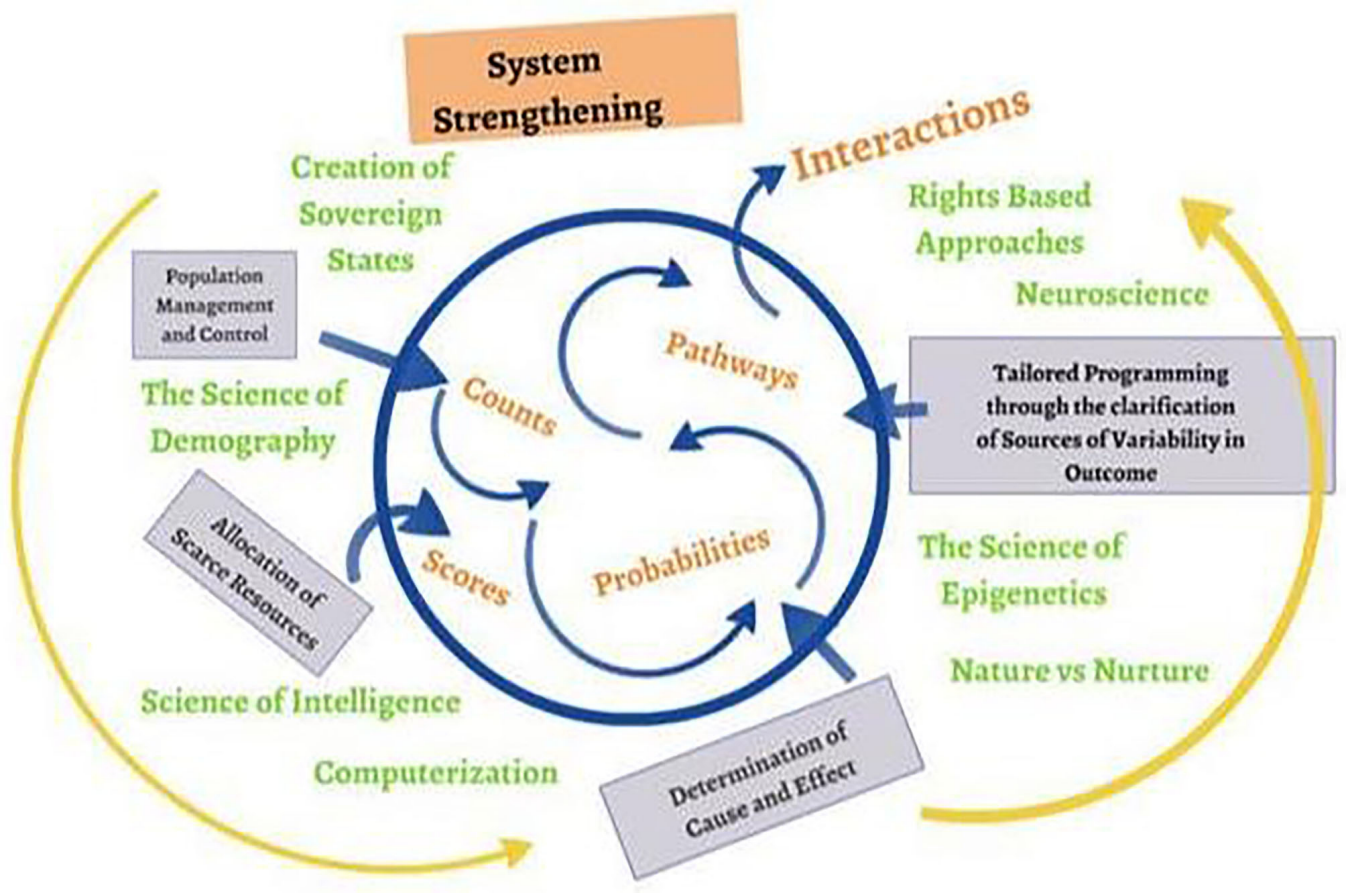
The evolution of monitoring, evaluation and learning (5–9).

The first influence was a reflection on measurement itself. Figure 2 summarizes the evolution of approaches to measurement that have supported decision-making at practice, program, and policy levels. Analytic methods, in the center, have developed out of, and contributed to, advances in science (including intelligence, epigenetics, and demography, indicated in green). Each new form of data has supplemented, not replaced, earlier measurement methods. Along with technical advances and theoretical shifts (also in green), monitoring and evaluation processes have strengthened implementations systems to meet changing, and ever more complex, service needs.

Being able to simultaneously track and regularly review multiple elements has supported program design to meet the specific needs of particular populations, enhancing effectiveness and increasing the “fit for purpose” of services, processes, and interventions. By incorporating additional sources of knowledge as they become available, the process of review and re-design has become even more informative. One of the contributing teams to the series, for example, has used social media to evaluate the quality of delivery, and applied both high- and low-tech methods to enhance community participation.

As advocated by United Nations agencies such as UNICEF, system strengthening develops out of shared decision-making by all contributors, and is informed by common learning (6). Such a (rights-based) framework requires the capacity to explore interactions. In contrast to the exploration of transactions, where influence is assumed to be unidirectional (A acts upon B, B acts) interactions acknowledge that in reality the response elicited from B will itself influence A, in a continuous cycle. A “feedback loop” is required to inform the consequences of an intervention, intended or otherwise, that influence the potential sustainability and scalability of interventions.

In summary, a recognition of the complexity of human behavior has generated the need for complex monitoring, evaluation and learning systems. The challenge is to apply these systems in practice given the constraints on resources of time, cost, and skill, often in challenging environments.

### The Struggle to Scale Interventions

While many innovations have had a measurable effect on early child development on a small scale, their effective transfer to larger scale delivery and across contexts remains a challenge (3, 4). Our experiences with ECD implementing organizations receiving support from, among others, the Saving Brains Program, have spurred the idea for more flexible MEL approaches to meet the challenges of scaling. One illustration from an organization in Uganda described the shift from the application of a pre-set fixed curriculum to the co-creation with the community of a parental training program. This more flexible approach, combining issues of interest and importance to the community with the science behind those issues, proved to be engaging for both the contributors and the delivery team, and was reported to be associated with high participation rates and quality of delivery. The necessity to adapt to new settings while also maintaining quality at scale may require both flexibility and rigor. The methods applied must also be recognized as valid. This last point is crucial if implementing teams wish to ensure the sustainability of their innovation.

### The Measurement for Change Team

Discussions of effective assessment^1^ brought the coordinators of this special issue together, and the role we play is to stimulate further discussion and to synthesize the ideas generated with the lessons learned from the translation of these ideas into practice. This multidisciplinary team comes from a variety of professional backgrounds, including implementation, research, MEL, as well as human rights advocacy and action, and includes the five authors of this paper.

## Decide

### Sharing and Developing the Measurement for Change Approach

The coordinating team agreed on a strategy to stimulate a critical review of the utilization of MEL in program design, implementation and decision-making within the ECD implementation community. Discussions were to focus around the articulation of a rigorous, systematic, but non-prescriptive, approach to measurement and scaling called “Measurement for Change.”

The initial approach was built around four key principles. These were that decisions should be continuously evidence-informed (Evidence), that all involved in the program should contribute to and benefit from the learning (Inclusion), that measurement processes should be responsive to the cyclical nature of change (Interactions) and that programs should be responsive to individual needs (Heterogeneity). The approach was to be reviewed and revised through a series of workshops and webinars attended by an invited audience of ECD practitioners and research groups. The culmination of these conversations is the current series of papers, detailing how these practitioners have used MEL to shape their decision-making. Through this series of articles, we intend to share the details of “Measurement for Change” more widely, what it is intended to achieve and how it has been applied in practice. This series will also act as an invitation for others to contribute to the continued evolution of this approach.

## Implementation

Here, we describe three events where the Measurement for Change approach was discussed with ECD practitioners, and how these discussions led to revision of the approach.

### Utrecht University Workshop

Invitees were drawn from implementation teams focused on early childhood development in South America, Europe, Africa, and Asia, supported by the Saving Brains Program and other associated early childhood programs.^2^ All workshop contributors (hereafter: contributors^3^) work in the field of early childhood development in low resource settings, with extensive experience of project design, research and implementation, active involvement in monitoring, evaluation, and the analysis of their programs. To bring this group together, a call for papers was circulated and the 21 responding organizations were invited to attend a writers' workshop on September 17 and 18, 2019 at Utrecht University, the Netherlands. Contributors were introduced to the concept and intention of Measurement for Change and were invited to reflect on this approach and its principles as they explored their own MEL systems. Exploration occurred through a series of detailed presentations, activities and focused discussions. Contributors presented practical examples of how data guided their decision-making, leading to re-design, adaptations and improvements in implementation and/or positioning for scaling of the intervention. The common challenges and lessons learned in implementation and scaling, as well as practical solutions, were captured and used to revise the Measurement for Change approach (1).

#### Reflections and Redesign

The contributors valued being part of a learning network through which they could explore how other organizations address measurement challenges. They drew attention to the large amounts of data collected during the design and implementation stages of a program. While this information is crucial to decision-making, it is rarely published, and therefore not openly available to inform others about essential adaptation and implementation processes. The contributors collectively defined the need for systematic documentation of the innovative use of data to contribute to global knowledge of effective delivery mechanisms.

The Measurement for Change approach, and the initial approach that was shared, resonated with the practitioners. Limitations in how contributors related the principles to their own practices reflected a need to articulate these principles more concretely. A mind map exercise of the common themes discussed drew attention to the regularity with which the word “flexibility” was mentioned. Contributors spoke of a flexible and growth mindset that was needed to apply the overall approach, and the individual principles. It was from this discussion that the concept of flexibility^4^ was added to the approach.

For the funding community to accept the need for extended periods of formative research within a fixed term funding cycle, contributors recognized the important advocacy role of those funders who already recognized the need for flexibility in the scaling process. They also recognized the need for published evidence of the effect of flexibility in effectively transitioning to scale. However, they questioned whether narratives reporting on the process, rather than the outcomes; and narratives that documented iterations in program content, rather than fixed intervention protocols, would be accepted for publication by journals. Without the support of journals, there would be significant limitations to sharing these experiences and the knowledge they generate.

### Wardha Workshop

In March 2020, at the “One Health” conference in Wardha, India, the ECD-track focused on the application of Measurement for Change amongst ECD implementation and research groups. The Datta Meghe Institute of Medical Sciences in India organized the workshop and the Saving Brains Program was invited to facilitate. The Wardha workshop contributors were drawn from groups delivering a variety of ECD-programs in India. Building upon the experience of the Utrecht University workshop, the Measurement for Change approach was more tightly embedded in examples of the application of the now expanded, set of principles. After introduction to the Measurement for Change approach, contributors were invited to present the measurement and data use systems of their own programs and to reflect on the contribution of these systems to supporting effective delivery mechanisms. Clinic group discussions that followed these presentations explored the capacity to apply the different principles and their contribution to effective program delivery. In the final session, contributors reflected on which principle was the least well-addressed in their projects, and how they will incorporate this principle, and its measurement, into their projects.

#### Reflections and Redesign

Workshop contributors recognized how the issues of complexity can be addressed using the Measurement for Change approach and valued the opportunity to apply it to their projects. The contributors reported this helped clarify the approach and enabled an adequate modeling of self-reflective practice and experiential learning.

The contributors recognized that collaborative decision-making is also an evolutionary process. Another learning was the need for the Measurement for Change approach to address planning for sustainable scaling early in project cycles. The contributors' acknowledgment that respect for others runs at the heart of Measurement for Change, is through positioning these concepts as central to all aspirations. They also recommended that Measurement for Change should explore the utility and effect of each aspiration for effective delivery of interventions.

### Writer's Webinar

The advancements made in the articulation of the Measurement for Change approach were presented to contributors of the Utrecht University Workshop, through a writers' webinar held in March 2020. The webinar was designed to support the completion of the papers being prepared for the series by clarifying the structure of the contributors' Measurement for Change narratives. During the webinar, the 11 attending contributors provided examples of their narratives and the accompanying discussions helped to refine the content and provide guidance for contributors who were uncertain how to proceed with their manuscripts.

#### Reflections and Redesign

Contributors indicated that the examples shared provided clarity about how to present their narratives in a format appropriate for a scientific paper. Feedback from each discussion opportunity also added clarity to the articulation of the approach. For example, we defined the dynamic principle through the phrase “stop, think, act.” Contributors indicated that “one never actually stops.” This aspiration was adjusted accordingly, and now describes a process of being cognizant and responsive to events and opportunities as they occur.

### Nine-Month Evaluation Survey

In June 2020, the coordinating team developed an online survey containing eight questions about the Measurement for Change development process and shared this survey with the Utrecht University workshop contributors. The survey had an 80% response rate. The survey was designed to assess how contributors applied the Measurement for Change approach to their projects and to evaluate the development process. We inquired about the usefulness of each of the implementation steps, the contributors' learning's throughout the process and the extent to which contributors felt included in the development process.

#### Reflection and Redesign

The survey showed that contributors valued inclusion in the process. The contributors indicated that applying the Measurement for Change approach helped their decision-making processes. Furthermore, the contributors valued the opportunity to meet the other teams. The majority of the contributors were still (and hoped to remain) in contact with at least one other team from the workshop. Between 70 and 90% of the contributors found the Utrecht University Workshop, discussions and feedback from the workshop team, and the writers' webinar useful. The contributors' interest in remaining involved in the Measurement for Change coordinating team's ambition to further explore the effect of the approach, led to the design of a Measurement for Change website. The website^5^ provides a platform to applications of the Measurement for Change approach, provided by the Utrecht University workshop contributors, Wardha workshop contributors, and new Measurement for Change ambassadors.

## Evaluation

The 1-year preparation phase we have described in this paper, illustrates how each of the aspirations of Measurement for Change were addressed in meeting the objectives of this initiative. The series of practitioner papers in this special issue will provide the evidence of how a shift from measurement of change (with a focus on fixed term outcomes) to measurement for change (with a focus on effective translation over time, place and need) is applied in practice. An editorial paper will be prepared to synthesize the learning across these diverse examples.

We brought together innovators from across the globe in workshops and webinars and inspired them to apply the approach to their own thinking and practice. The collaborative process has provided confirmation that Measurement for Change as an approach has the potential to significantly strengthen the implementation process. Discussions have provided examples that include the contribution of an iterative process to adapting to change, the benefits to decision-making of multiple data sources, and of the important contribution of actors in the local context to sustainable scaling. The measurement of the direct effect of these processes will need to be the focus of future initiatives.

The rich discussions generated through the in-person and virtual meetings have added considerable clarity to the articulation and shared understanding of the Measurement for Change approach (1). We learned there is extensive interest in exploring in detail the implementation process, to reflect on that process, and to share the knowledge gained from that process. This is supported by the fact that, at the time of the 9-month survey, 17 teams still intended to submit a full manuscript despite their busy schedules. We also learned that implementation cannot be too tightly defined. Deadlines for the series had to be adjusted to fit the time it takes for these reflections to come together, and to adjust to the pressures on teams to respond to unanticipated events such as Covid-19.

The development of the Measurement for Change approach does not end here. It will continue to evolve as it responds to new insights and further scientific and technological advances. In planning this journey, we attempted to respond to individual needs by providing different forums for exploration and discussion. Contributors rated most highly the opportunity to meet, share and discuss experiences, challenges and lessons learned. The results highlighted the need for knowledge- and experience-sharing platforms (collective learning spaces) for implementers and the desire to remain part of the development process (co-creation).

In sum, the collaborative design has been essential to the achievement of our short-term objectives, as it will be to the continued development of the Measurement for Change approach. Through the publication of this series, and the proposed launch of a website forum, we intend to continue this journey to address the challenge of translating good ideas into effective sustainable and scalable practice.

## Data Availability Statement

The original contributions presented in the study are included in the article/supplementary material, further inquiries can be directed to the corresponding author/s.

## Author Contributions

LH led the writing, editing, but substantial written, and oral contributions were provided by all authors. All authors contributed equally to the development of the process presented in this Perspective.

## Conflict of Interest

The authors declare that the research was conducted in the absence of any commercial or financial relationships that could be construed as a potential conflict of interest.

## Footnotes

^1^Dubai 2019 The Saving Brains Transition to Scale Workshop, organized by Grand Challenges Canada and funded by a private philanthropy.

^2^https://www.grandchallenges.ca/programs/saving-brains/.

^3^Contributors to the Utrecht University workshop are the main group of contributors we follow through this process (the Writers' webinar, the evaluation survey, the paper series). Contributors to the Wardha Workshop are a different group of academics and ECD-practitioners.

^4^The flexibility-principle was renamed as the “Dynamic” principle.

^5^The website, as it is under construction, has limited visibility. It is hoped to make it open access in 2021.
